# 901 Documentation of a Primary Care Physician Is Associated with Higher 90-day Burn Center Readmission

**DOI:** 10.1093/jbcr/iraf019.432

**Published:** 2025-04-01

**Authors:** Elliott Yee, Darby Little, Barbara Haas, Stephanie Mason

**Affiliations:** University of Toronto; University of Toronto; University of Toronto; Ross Tilley Burn Centre, Sunnybrook Health Sciences Centre

## Abstract

**Introduction:**

Burn survivors may require readmission after burn center discharge due to complications or for reconstruction. Primary care physicians (PCPs) may play an important role in the post-discharge care of burn patients, both in managing complications and facilitating burn center readmission when patient needs exceed community resources. The objective of this study was to assess the relationship between access to a community PCP and burn center readmission following initial discharge, hypothesizing that PCP access would be associated with a lower likelihood of readmission.

**Methods:**

This retrospective cohort study included patients ≥ 15 years old admitted to a regional burn center from 2009–2023 who were alive at discharge. Variables were obtained from chart review and burn registry data. The exposure under study was the presence of a documented PCP in the inpatient record during initial burn center admission. The primary outcome was readmission to the same burn center for any reason within 90 days of discharge. We evaluated whether having a documented PCP during the initial burn-related admission was independently associated with likelihood of readmission using a multivariable logistic regression model adjusted for age, gender, total body surface area (TBSA) burned, inhalation injury, length of stay, operating room visits, complications, comorbidity, disability, smoking, and discharge disposition.

**Results:**

Of 2,244 patients, 67% had a documented PCP and 5.6% were readmitted to the burn center within 90 days. Mean age was 46 years, 71% were male, and mean TBSA burned was 10%. Patients who were older, female, or had medical comorbidity were more likely to have a PCP documented. PCP documentation was independently associated with a greater likelihood of readmission (OR 1.61, 95% CI 1.05-2.47). More operating room visits or a major complication during the initial admission were also associated with higher likelihood of readmission.

**Conclusions:**

Burn survivors with a documented PCP were more likely to be readmitted to the burn center within 90 days, suggesting that PCP access facilitates escalation of care for burn survivors in the community. As burn center care is highly centralized, PCPs may play an important role in triaging post-discharge complications in the outpatient setting and coordinating readmission to burn centers. These findings highlight the importance of PCP documentation during hospital admission to ensure that patients receive appropriate care after discharge.

**Applicability of Research to Practice:**

The findings of this study can inform interventions that improve communication between inpatient burn clinicians and outpatient PCPs and can guide future studies examining post-discharge care pathways for burn survivors.

**Funding for the Study:**

N/A

